# Solution structure of a *Plasmodium falciparum *AMA-1/MSP 1 chimeric protein vaccine candidate (PfCP-2.9) for malaria

**DOI:** 10.1186/1475-2875-9-76

**Published:** 2010-03-18

**Authors:** Heng Peng, Yunfei Hu, Aiguo Zhou, Changwen Jin, Weiqing Pan

**Affiliations:** 1 Department of Pathogenic Biology and State Key Laboratory of Medical Immunology, Second Military Medical University, 800 Xiang Yin Road, Shanghai 200433, China; 2 Beijing Nuclear Magnetic Resonance Center, College of Life Sciences, Peking University, Beijing 100871, China

## Abstract

**Background:**

The *Plasmodium falciparum *chimeric protein PfCP-2.9 is a promising asexual-stage malaria vaccine evaluated in clinical trials. This chimeric protein consists of two cysteine-rich domains: domain III of the apical membrane antigen 1 (AMA-1 [III]) and the C-terminal region of the merozoite surface protein 1 (MSP1-19). It has been reported that the fusion of these two antigens enhanced their immunogenicity and antibody-mediated inhibition of parasite growth *in vitro*.

**Methods:**

The ^15^N-labeled and ^13^C/^15^N-labeled PfCP-2.9 was produced in *Pichia pastoris *for nuclear magnetic resonance (NMR) structure analysis. The chemical shift assignments of PfCP-2.9 were compared with those previously reported for the individual domains (i.e., PfAMA-1(III) or PfMSP 1-19). The two-dimensional spectra and transverse relaxation rates (*R*_2_) of the PfMSP1-19 alone were compared with that of the PfCP-2.9.

**Results:**

Confident backbone assignments were obtained for 122 out of 241 residues of PfCP-2.9. The assigned residues in PfCP-2.9 were very similar to those previously reported for the individual domains. The conformation of the PfMSP1-19 in different constructs is essentially the same. Comparison of transverse relaxation rates (*R*_2_) strongly suggests no weak interaction between the domains.

**Conclusions:**

These data indicate that the fusion of AMA-1(III) and MSP1-19 as chimeric protein did not change their structures, supporting the use of the chimeric protein as a potential malaria vaccine.

## Background

Malaria is one of the most serious life-threatening tropical diseases in the world. Because of the rapid spread of drug-resistant parasites and insecticide-resistant mosquitoes [1-4], new tools for control malaria are urgently needed. The 200-kDa merozoite surface protein-1 (MSP 1) and the apical membrane antigen (AMA-1) of *Plasmodium falciparum *are attractive candidates for malaria vaccines [5-9]. These two antigens are located on the merozoite surface and have been proposed to play a role in the invasion process [10-15]. A portion of the MSP1 targeted by protective immunity antigen has been mapped to the 19 kDa carboxy-terminal region (MSP1-19) which contains two tandem repeat epidermal growth factor (EGF)-like domains while the most C-terminal of the disulphide-bonded domains in AMA-1 (Domain III) was also a target for inhibitory antibodies isolated from malaria patients [16-20].

A chimeric protein (PfCP-2.9) was constructed comprising the sequences of both AMA-1(III) and the MSP 1-19 from *P. falciparum *[21]. The two proteins were fused via a hinge encoding a Gly-Pro-Gly motif repeat, and a secreted form of the PfCP-2.9 protein was expressed in *Pichia pastoris*. The fusion enhanced product yield, immunogenicity, and antibody-mediated inhibition of parasite growth *in vitro*. Sera from rabbits and rhesus monkeys immunized with the chimeric antigen almost completely inhibited parasite growth. Two phase I clinical trials of this vaccine candidate formulated in Montanide ISA 720 were completed recently, demonstrating the safety, tolerability, and immunogenicity of the vaccine in humans [22,23].

The PfCP-2.9 chimeric protein contains 18 cysteine residues, six of which are located in AMA-1(III) region and the rest in the MSP 1-19 region, that form nine intramolecular disulfide bonds. Protective immunity conferred by this vaccine candidate was shown to be dependent on its disulfide backbone-based conformation. Immune sera containing reduced and alkylated PfCP-2.9 did not inhibit parasite growth, indicating that induction of the growth-inhibitory response required proper folding of this chimeric protein [21]. Therefore, it is necessary to characterize the structure of the fusion protein. In the present study, the ^15^N- and ^15^N/^13^C-labeled PfCP-2.9 protein were expressed in *P. pastoris *to determine its solution structure.

## Methods

### Reagents

^15^NH_4_SO_4 _and ^13^C-D-glucose was purchased from Cambridge Isotope Laboratories (Andover, MA, USA). ^13^C-methanol was purchased from Spetra (Columbia, MD, USA).

### Preparation of ^15^N-labeled PfCP-2.9

The stock *P. pastoris *strain [21] expressing PfCP-2.9 with C-terminal 6 × His tags was streaked on a YPD agar plate (1% Yeast extract, 2% Peptone, 2% Glucose, 2% agar) containing the antibiotic G418 (0.25 mg/ml). Clones were incubated in 150 ml BMGY medium (1.34% yeast nitrogen base [YNB] without ammonium sulfate and amino acids, 1% yeast extract, 2% peptone, 1% glycerol, 4 × 10^-5^% biotin, and 100 mM potassium phosphate [pH 6.0]) and grown to an optical density of approximately 20 at 600 nm (OD_600_). The cells were then transferred into 3L of ^15^N salt base medium (2.67% [v/v] H_3_PO_4 _(85%), 0.0894% CaSO_4_, 1.52% K_2_SO_4_, 1.49% MgSO_4_· 7H_2_O, 0.413% KOH, 4% glycerol, 0.4% [v/v] PTM1 salts, 0.9% [NH_4_]_2_SO_4_) in a 5-L fermenter. OD_600 _reached 75 after 21 hr, and 180 g methanol was then added to induce expression of the chimeric protein. After 19 hr, the culture was centrifuged at 6000 × g for 20 min at 4°C, and the supernatant was collected for protein purification.

The target protein was purified by Ni-NTA agarose column (Qiagen, Hilden, Germany) affinity purification. Ten milliliters of Ni-NTA agarose was equilibrated with the loading buffer (50 mM NaH_2_PO_4_, 300 mM NaCl, pH 8.0) without a reducing agent. The cell culture supernatant was then applied to the column at 1 ml/min, and flow through was collected for analysis. The column was washed with 60 ml loading buffer followed by 120 ml washing buffer (50 mM NaH_2_PO_4_, 300 mM NaCl, 20 mM imidazole, pH 8.0). Bound proteins were eluted with 250 mM imidazole. Eluted proteins were concentrated into 1 ml using an Amicon Ultra-15 centrifugal filter unit (10 kDa, Millipore, Billerica, USA).

The NMR sample was prepared in a buffer containing 20 mM sodium phosphate in 90% H_2_O/10% D_2_O (pH 7.4). The sample was argon-flushed.

### Preparation of ^13^C/^15^N-labeled PfCP-2.9

The stock *P. pastoris *strain was streaked on a YPD agar plate and grown in 3 L of ^15^N salt base as described above. The cell density reached OD_600 _of 60 after 17 hr, and 5 g of ^13^C-D-glucose was then added. After 2 hr, 50 g of ^13^C-methanol was added to induce chimeric protein expression. After 6 hr, the culture was centrifuged at 6,000 × g for 20 min at 4°C, and the supernatant was collected for protein purification.

The purification and NMR sample preparation of the target protein was same as for ^15^N-labeled PfCP-2.9. The final volume was 1.2 ml. Protein concentration was 10 mg/ml as determined by the Bradford method, and purity was > 90% as assessed by SDS-PAGE.

### Preparation of ^15^N-labeled PfMSP1-19

The stock *P. pastoris *strain was streaked on a YPD agar plate containing 0.25 mg/ml G418, which was preserved in laboratory. A clone was incubated in 500 ml BAGY medium (0.34% YNB without ammonium sulfate and amino acids, 1% glycerol, 0.5% (NH_4_)_2_SO_4_, 4 × 10^-5^% biotin, and 100 mM potassium phosphate [pH 6.0]) and grown to OD_600 _of approximately 10. Cells were collected by centrifugation and transferred into 500 ml of ^15^N BMM medium (0.34% YNB without ammonium sulfate and amino acids, 0.5% methanol, 0.5% (NH_4_)_2_SO_4_, 4 × 10^-5^% biotin, and 100 mM potassium phosphate [pH 6.0]). Every 12 hr, 1.25 ml of methanol was added to the culture medium. After 72 hr, the culture was centrifuged at 6,000 × g for 20 min at 4°C to collect the supernatant for protein purification. The purification process and NMR sample preparation was the same as for PfCP-2.9.

### Nuclear magnetic resonance data collection and analysis

The nuclear magnetic resonance (NMR) spectra were acquired at 25°C with a Bruker Avance 500 (with a CryoProbe) and 800 MHz spectrometers, equipped with four RF channels and a triple-resonance probe with pulsed field gradients. The three-dimensional (3D) HNCA, HNCACB, CBCA(CO)NH, HNCO, and HN(CA)CO experiments were carried out for the backbone assignments. HBHA(CO)NH, (H)CC(CO) NH, CC(CO)NH, and ^15^N-TOCSY-HSQC experiments were performed for side-chain assignments [24]. 3D ^15^N-edited NOESY-HSQC spectrum (mixing time, 100 ms) was performed to confirm the chemical shift assignments. All spectra were processed using the software package NMRPipe [25] and analysed with the programme NMRView [26].

### Backbone ^15^N transverse relaxation measurements

The backbone ^15^N transverse relaxation experiments of the PfCP-2.9 and MSP 1-19 domain were recorded [27] on a Bruker AVANCE 800-MHz NMR spectrometer at 25°C. For both samples, 512 (^1^H) and 70 (^15^N) complex data points were collected with 48 transients per increment and a recycle delay of 3.0 s. The delays were 4 (×2), 32, 60, 100, 160 ms for ^15^N-labeled MSP 1-19, and 4 (×2), 12, 28, 48, 72, 100, 160 ms for ^15^N-labeled PfCP-2.9. The transverse relaxation rate constants (*R*_2_) were obtained by fitting the peak intensities to a single exponential function by nonlinear least-squares method using MATLAB [28].

## Results

### Chemical shift assignments of PfCP-2.9

**Figure 1:**
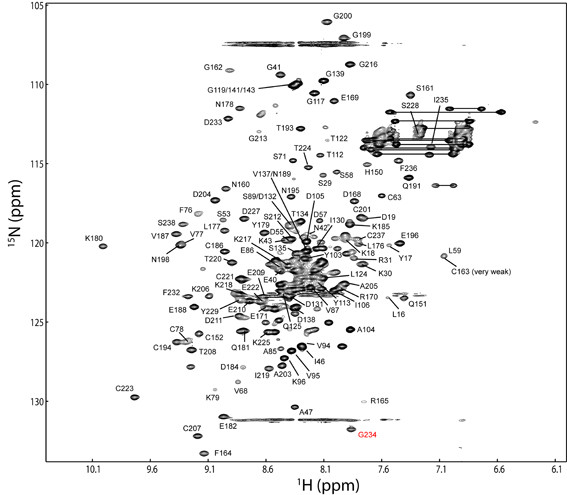
**The 2D ^15^N-edited HSQC spectrum showing the backbone assignments of PfCP-2.9**. The assigned backbone amides of PfCP-2.9 are labeled with the one-letter amino acid codes and are numbered according to the sequence of PfCP-2.9. The residue G234 (labeled in red) is folded in the spectrum.

**Figure 2:**
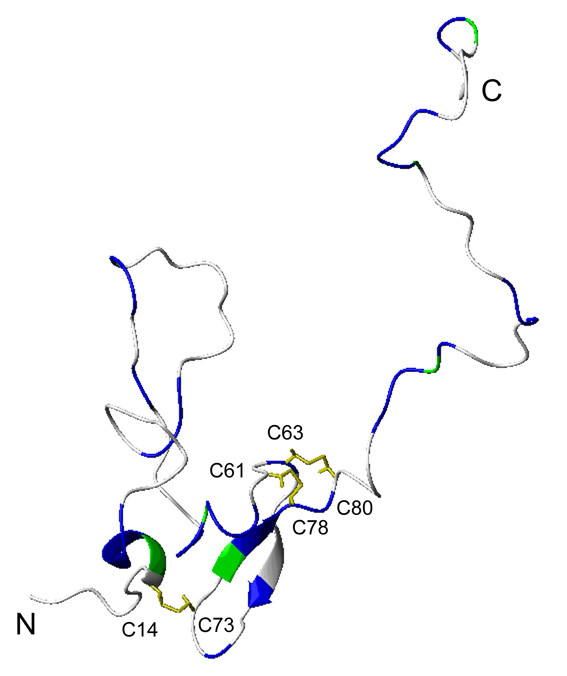
**Mapping of the assigned residues onto the AMA1 domain III structure**. The residues with assigned backbone amides are blue. The proline residues (which have no amides) and residues without backbone assignments but with C^α ^and C^β ^atoms that could be assigned are green. The disulfide bridges are shown, and the cysteine residues are labeled. The residues are numbered according to the sequence of PfCP-2.9. The structure corresponds to the first conformer of the NMR structures of AMA1 domain III (PDB code 1HN6). The figure was generated by MOLMOL.

The chemical shift assignments of PfCP-2.9 were performed based on the triple-resonance NMR spectra aided by the previously reported NMR assignments for the PfAMA-1(III) domain (Biological Magnetic Resonance Data Bank [BMRB] entry 4787) and PfMSP1-19 domain (BMRB entry 4437) [29,30]. Backbone assignments were obtained for 39 out of 116 residues of the PfAMA-1(III) domain, 12 out of 28 residues of the hinge region, and 71 out of 97 residues of the PfMSP1-19 domain. Side-chain chemical shifts were also partially obtained. As shown in the 2D ^15^N-edited heteronuclear single quantum coherence (HSQC) spectrum of PfCP-2.9 (Figure 1), most of the well-dispersed peaks were assigned. The residues that could not be unambiguously assigned were clustered in the central region of the spectrum due to severe overlap. For the PfAMA-1(III) domain, the residues with assigned backbone amides and C^α ^and/or C^β ^atoms were Leu16-Asp19, Ser29-Arg31, Gly42-Lys44, Ile46-Ala47, Ser53-Asp55, Asp57-Leu59, Cys63, Val68, Ser71, Phe76-Cys78, Ala85-Val87, Ser89, Val94-Lys96, Tyr103-Ile106, and Thr112-Tyr113. Additional chemical shift assignments of the C^α ^and/or C^β ^atoms (and in most cases H^α ^and H^β ^atoms) were obtained for residues Ser15, Lys56, Phe75, Thr88, Glu102, and Pro111. The mapping of these assigned residues onto the NMR structure of the PfAMA-1(III) domain (PDB code 1HN6) is shown in Figure 2, which indicates that the assigned residues are spread over different regions of this domain, including both well-structured and disordered regions. In particular, many residues in the core structural region were assigned.

**Figure 3:**
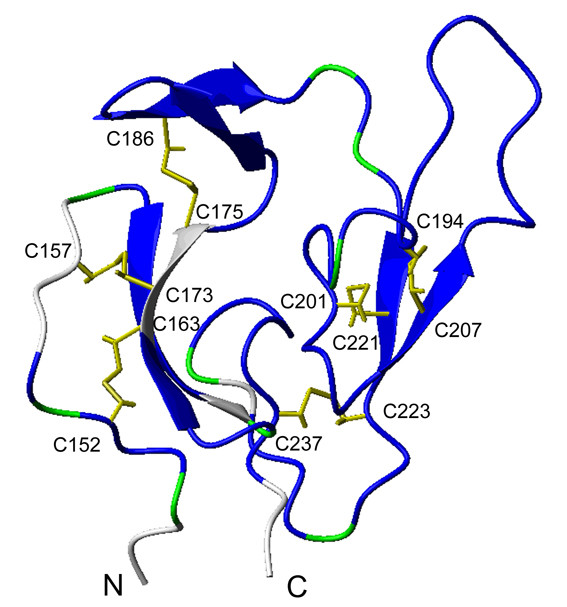
**Mapping of the assigned residues onto the MSP1-19 domain structure**. The residues with assigned backbone amides are blue. The proline residues (which have no amides) or residues without backbone assignments but with C^α ^and C^β ^atoms that could be assigned are green. The disulfide bridges are shown, and the cysteine residues are labeled. The residues are numbered according to the sequence of PfCP-2.9. The structure corresponds to the first conformer of the NMR structures of MSP 1-19 domain (PDB code 1CEJ). The figure was generated by MOLMOL.

For the PfMSP1-19 domain, the residues with assigned backbone amides as well as C^α ^and/or C^β ^atoms are His150-Cys152, Lys154, Asn160-Arg165, Asp168-Glu171, Leu176-Asn189, Gln191, Thr193-Glu196, Asn198-Lys225, Asp227-Tyr229, and Phe232-Ser238. Additional chemical shift assignments of the C^α ^and C^β ^atoms (and in most cases H^α ^and H^β ^atoms) can be obtained for residues Gln149, Val153, Gln159, Leu167, Pro190, Pro192, Asn197, Pro226, and Leu231. The mapping of these assigned residues onto the NMR structure of the MSP 1-19 domain (PDB code 1CEJ) is displayed in Figure 3, which shows that the majority of the residues in this domain were assigned.

### Comparison of the assigned chemical shifts of PfCP-2.9 with those of PfAMA-1 (III) and Pf MSP1-19 domains

**Figure 4:**
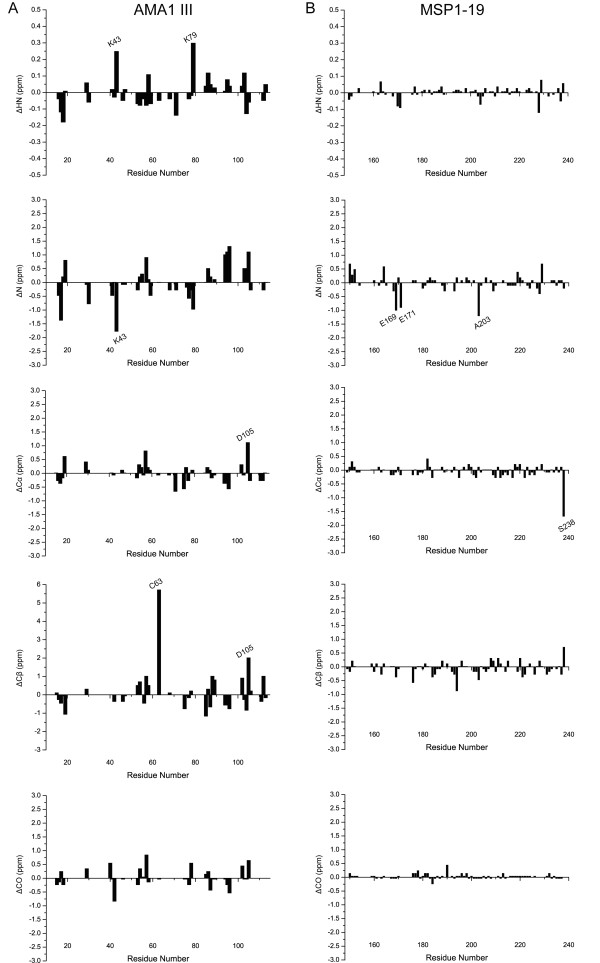
**Chemical shift comparison between PfCP-2.9 and the individual AMA1 III and MSP 1-19 domains**. (A) Chemical shift differences for the H^N^, N, C^α^, C^β^, and CO atoms between the AMA1 III domain in PfCP-2.9 and the AMA1 III domain alone (BMRB entry 4787). (B) Chemical shift differences for the H^N^, N, C^α^, C^β^, and CO atoms between the MSP 1-19 domain in PfCP-2.9 and the MSP 1-19 domain alone (BMRB entry 4437). The residues are numbered according to the sequence of PfCP-2.9. The figure was generated by Origin 8.0.

As shown in Figure 4, nearly all of the assigned residues in PfCP-2.9 display chemical shifts highly similar to those reported for the individual domains PfAMA-1(III) and PfMSP1-19 [29,30]. For the residues assigned in PfAMA-1 III domain, the majority show the following chemical shift differences: |ΔH^N^| < 0.2 ppm, |ΔN| < 1.5 ppm, |ΔC^α^| < 1.0 ppm, |ΔC^β^| < 1.5 ppm, and |ΔCO| < 1.0 ppm. Only a few atoms show relatively large chemical shift differences between the present results and previously published assignments of AMA1 III (BMRB entry 4787) [29]. These include the H^N ^atoms of Lys43 and Lys79, the backbone ^15^N atom of Lys43, the Cα atom of Asp105, and the Cβ atoms of Cys63 and Asp105. The difference might be due to the fact that in the NMR study of AMA1 III domain alone was performed using refolded protein under acidic (pH 3.4) buffer condition [29], while in the present study neutral (pH 7.4) buffer condition was used.

For the residues assigned in the PfMSP1-19 domain, the majority show only small chemical shift differences: |ΔH^N^| < 0.1 ppm, |ΔN| < 1.0 ppm, |ΔCα| < 0.5 ppm, |ΔCβ| < 1.0 ppm, |ΔCO| < 0.5 ppm. Few atoms show bigger chemical shift differences between the current results and previously published assignments of PfMSP1-19 (BMRB entry 4437) [30]; these include the backbone ^15^N atoms of Glu169, Glu171 and Ala203, and the Cα atom of Ser238. The Glu169 and Glu171 residues are acidic residues, whereas the Ala203 residues are located between two Asp residues. The larger ^15^N chemical shift differences for these three residues probably result from the pH differences of buffers used in the two studies (buffer pH used in the previous study of the PfMSP1-19 domain was 6.5).

### Spectral comparison between PfCP-2.9 and MSP 1-19 domain

**Figure 5:**
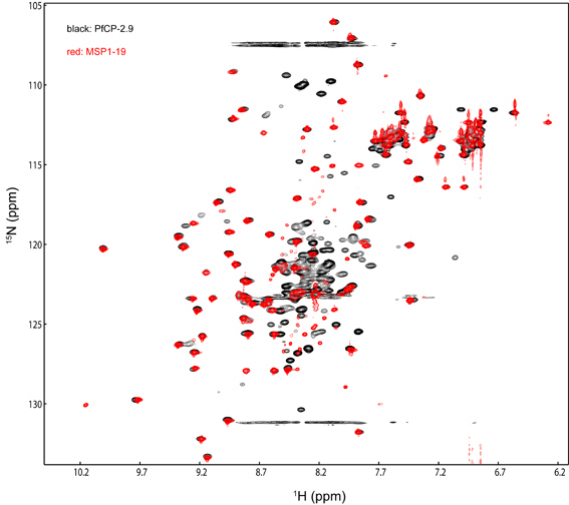
**Overlay of 2D ^15^N-edited HSQC spectra of PfCP-2.9 and MSP 1-19**. The spectrum of PfCP-2.9 is shown in black and spectrum of MSP 1-19 alone is shown in red.

**Figure 6:**
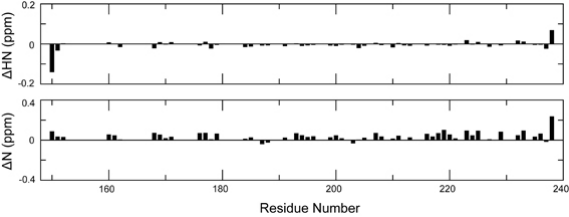
**Chemical shift comparison between PfCP-2.9 and MSP 1-19**. Chemical shift differences of backbone H^N ^atoms (upper panel) and N atoms (lower panel) between MSP 1-19 and PfCP-2.9 are shown. The chemical shift differences were calculated by subtracting the chemical shift values for each residue in the MSP 1-19 domain in PfCP-2.9 from those in MSP 1-19 alone. The residues are numbered according to the sequence of PfCP-2.9. The figure was generated by Xmgrace.

To provide further evidence that the structures of the two domains remain unchanged in the chimeric protein PfCP-2.9, the PfAMA-1(III) and PfMSP1-19 domains were expressed separately in *P. pastoris *and dissolved in the same buffer as PfCP-2.9. Unfortunately, the ^15^N-edited HSQC spectrum of the ^15^N-labeled PfAMA-1(III) domain indicated that the separately expressed protein domain was not properly folded; however, the ^15^N-edited HSQC spectrum of ^15^N-labeled PfMSP1-19 domain showed well-dispersed peaks, demonstrating the protein had folded well. The overlay of the HSQC spectra of PfMSP1-19 and PfCP-2.9 (Figure 5) demonstrated that the structure of the PfMSP1-19 domain in PfCP-2.9 is the same as PfMSP1-19 expressed alone. The chemical shift differences (Figure 6) were within experimental errors for all peaks, with the exception of His150 and Ser238. His150 is preceded by the linker sequence in PfCP-2.9, whereas it is close to the N-terminus in PfMSP1-19. The residue Ser238 is close to the C-terminus of both proteins and could be easily affected by slight condition differences between samples (e.g., differences in purity). Moreover, no any signal disappearance was observed by comparing the two spectra. These data strongly demonstrated that the conformation of PfMSP1-19 domain in the fusion protein is identical to PfMSP1-19 domain alone.

### Comparison of transverse relaxation rates between PfCP-2.9 and PfMSP1-19 domain

The lack of change in chemical shifts of the PfMSP1-19 domain when expressed as a part of the fusion protein PfCP-2.9 excludes the possibility of strong interactions between PfMSP1-19 and PfAMA-1(III) domains; however, weak interactions between the two domains are possible. If weak interactions between the two domains exist, one or both of the following phenomena were expected to seen: (1) chemical shift differences for residues at the interaction surface; (2) line broadening due to chemical/conformational changes.

Neither chemical shift changes between the two spectra nor peak disappearance was observed; therefore, the transverse relaxation rates *R*_2 _of the two samples were measured to further investigate the possibility of line broadenings. For the data analysis of PfCP-2.9, only those residues present in PfMSP1-19 were analysed. The peaks that overlapped were excluded from the analysis. Further, residues His150, Glu171, Asp184, Gly213, Ser228, and Ser238 showed weak signals and poor relaxation curves in both samples, thus were also removed from the final analysis. The *R*_2 _values of a total of 36 residues (Cys152, Asn160-Gly162, Phe164, Asp168-Glu169, Leu177-Lys180, Cys186-Glu188, Asn191, Thr193-Glu196, Gly199-Gly200, Ala203-Asp204, Lys206-Thr208, Gly216, Ile219-Thr220, Cys223-Thr224, Asp227, and Asp233-Phe236) were finally obtained.

**Figure 7:**
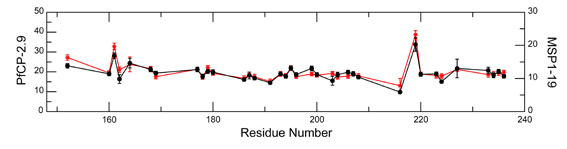
**Comparison of transverse relaxation rates (R_2_) between PfCP-2.9 and MSP 1-19**. Overlay of the *R*_2 _values of MSP 1-19 (red stars) alone and the MSP 1-19 domain in PfCP-2.9 (black circles). The scale of the *R*_2 _values for PfCP-2.9 is on the left, and the scale for MSP 1-19 is on the right. The residues are numbered according to the sequence of PfCP-2.9. The figure was generated by Xmgrace.

The *R*_2 _values of these residues were compared between PfCP-2.9 and PfMSP1-19. The *R*_2 _values were overall higher for residues in PfCP-2.9 compared with those in PfMSP1-19, which is due to the higher molecular weight and slower molecular tumbling of PfCP-2.9. By overlaying the two datasets (Figure 7), the trend of *R*_2 _values over residue number is essentially the same for the two samples. No significant increase of transverse relaxation rates were observed for any region. This result indicates that no residue chemical/conformational changes occurred in the PfMSP1-19 domain of PfCP-2.9, suggesting that the PfMSP1-19 domain is not likely to be involved in weak interactions with the AMA-1(III) domain or the linker region.

## Discussion

It has been reported that a panel of monoclonal antibodies recognizing conformational epitopes on PfMSP1-19 interacted with the chimeric protein with reduced sensitivity [21]. Of these monoclonal antibodies, mAb2.2 and 12.8 bind to the first EGF domain of PfMSP1-19, whereas mAb111.2 requires the presence of both EGF-like domains [31]. The mAb12.8 is an inhibitory antibody while mAb 2.2 and mAb 1E1 are blocking antibodies [32,33]. These data indicated that the critical epitopes were retained after fusion of the two domains into one molecule; however, correct folding of the PfAMA-1(III) portion and other epitopes on the PfMSP1-19 and potential interactions between the two domains was not known.

In the present study, the chemical shift assignments of PfCP-2.9 was compared with those previously reported for individual domains and found that the structures of the two domains were unchanged. Further, comparison of the 2D spectra of PfMSP1-19 with the domain in the PfCP-2.9 also demonstrated that the conformation of the domain is essentially the same, while the PfAMA-1(III) domain showed better folding in the chimeric protein than it did alone.

Comparison of transverse relaxation rates *R*_2 _between the PfMSP1-19 domain in PfCP-2.9 and the domain alone strongly suggests no weak interactions between domains. Taken together, these data suggested that the structures of the PfAMA-1(III) and PfMSP1-19 domains were not altered by fusion into the chimeric protein.

Crystallization the chimeric protein were unsuccessful despite various efforts. Therefore, ^15^N-labeled and ^13^C/^15^N-labeled PfCP-2.9 were expressed in *P. pastoris *for NMR structure analysis. The backbone chemical shift assignments can be obtained for a reasonable portion of the residues in PfCP-2.9. By comparing with the previously reported studies of the two domains alone, high similarity of chemical shifts was observed. Relatively larger differences were observed for only a small number of residues, which may be due to different experimental conditions (e.g., buffer pH and temperature). In fact, almost all the residues that showed significant chemical shift changes in PfAMA-1(III) were charged residues, with the exception of Cys63, which is near charged residues according to the NMR structure of PfAMA-1 (III). In addition, PfCP-2.9 was modified in the following ways: three glycosylation sites were removed by changing Asn to Gln, a hinge consisting of 28 residues was inserted between the two domains [21], and a 6×His-tag was added to its C-terminus. These modifications can also influence the chemical shift of nearby residues.

The PfCP-2.9 chimeric protein induces antibodies that inhibit parasite growth *in vitro*, providing additional evidence for the unchanged protein conformation. Moreover, both components of the chimeric protein are able to generate inhibitory antibodies against parasite growth, indicating that both domains fold correctly [21]. NMR findings in the present study were consistent with previous reports. The lack of interaction between the two domains greatly reduces the possibility that novel epitopes exist in the chimeric protein.

## Competing interests

The authors declare that they have no competing interests.

## Authors' contributions

HP participated in the study design, performed the experiments and data analysis and wrote the manuscript. YH performed the NMR experiment, analysed the NMR data and contributed to manuscript writing. AZ prepared the ^15^N-labled and ^13^C/^15^N-labled PfCP-2.9. CJ participated in study design, NMR data analysis, and contributed to manuscript writing. WP conceived of the study, participated in its design, data analysis, and contributed to manuscript writing and editing. All authors read and approved the final manuscript.
